# Ultrasound-Guided Clavipectoral Fascial Plane Block as a Stand-Alone Anesthetic Technique for Clavicle Fracture Management: A Case Report

**DOI:** 10.7759/cureus.60244

**Published:** 2024-05-13

**Authors:** Swati Vijapurkar, Gade Sandeep, Suresh Rajwade, Jakkireddy Sravani, Nimisha Cherunghattil

**Affiliations:** 1 Anaesthesiology, All India Institute of Medical Sciences, Raipur, IND; 2 Cardiac Anaesthesiology, All India Institute of Medical Sciences, Raipur, IND

**Keywords:** dexmedetomidine, ultrasound, clavicle fracture, analgesia, anesthesia

## Abstract

The clavipectoral fascial plane block (CFPB) that has been utilized for clavicle fracture surgeries and pain management is an emerging anesthetic technique. It has been previously used for postoperative pain management, but it can also be used as a stand-alone anesthetic technique for clavicle fracture management. Here we describe a case of a 20-year-old male who underwent open reduction and internal fixation (ORIF) with plating for a mid-shaft clavicular fracture under a CFPB as a sole anesthetic.

## Introduction

Clavicle fractures, which are frequently encountered in both the emergency department and the operating room, account for 2.6% of all fractures [1]. Providing regional anesthesia via peripheral nerve blocks can be a challenge to the anesthetist due to multiple innervations of the clavicle and the requirement of a combination of peripheral nerve blocks like cervical plexus block and brachial plexus block, which are associated with complications like phrenic nerve palsy and pneumothorax, respectively [2]. Hence, traditionally, general anesthesia has been the preferred anesthesia for clavicle fractures [3]. The clavipectoral fascial plane block (CFPB) that has been utilized for clavicle fracture surgeries and pain management is an emerging anesthesia technique. The clavipectoral fascia is a tough fascial sheet that fills the space between the clavicle and the pectoralis minor muscle and is deep into the clavicular head of the pectoralis major muscle [4]. Here we describe a case of a 20-year-old male who underwent open reduction and internal fixation (ORIF) with plating for a mid-shaft clavicular fracture under a CFPB as a sole anesthetic.

## Case presentation

**Figure 1:**
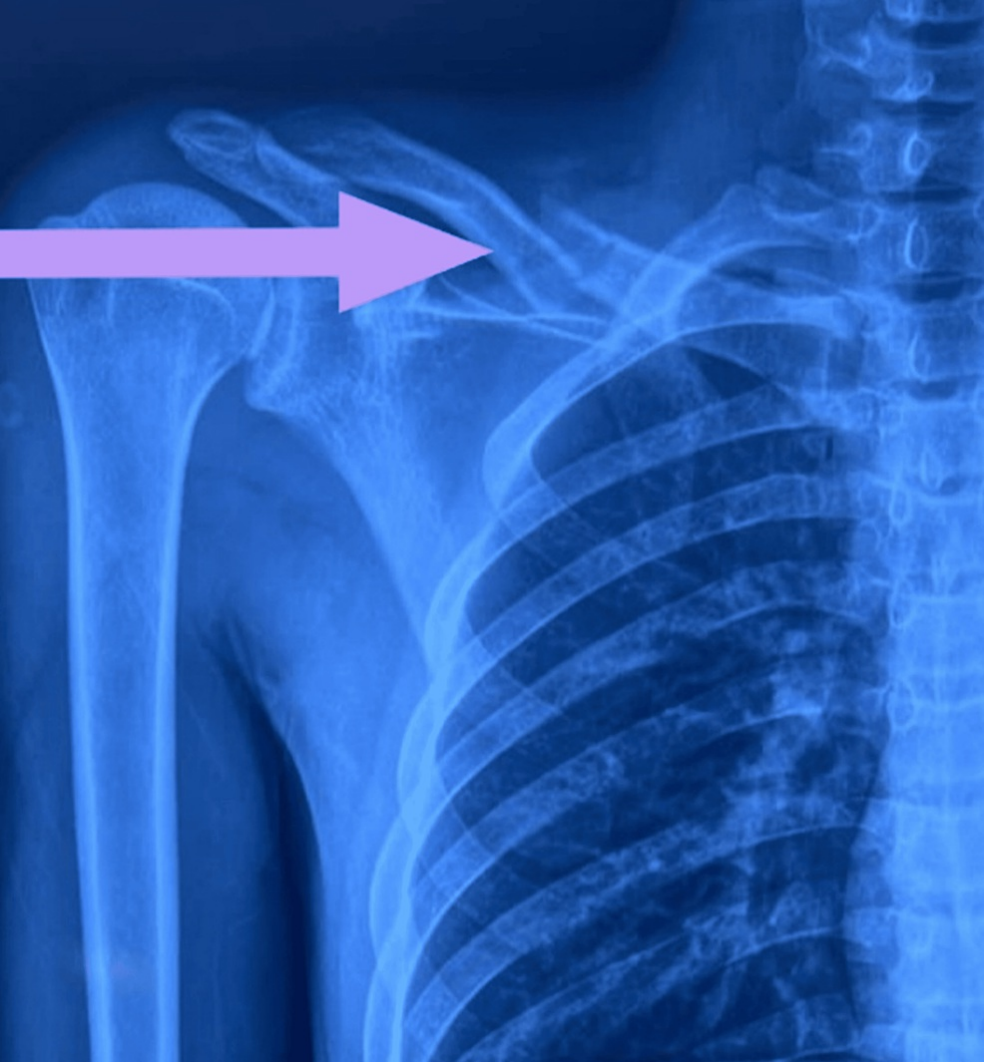
An X-ray shows a fracture of the mid-shaft of the clavicle.

A 20-year-old male patient weighing 65 kilograms and 174 cm in height presented to the emergency department with an alleged history of a road traffic accident, following which he sustained an injury to his right shoulder. The patient did not have any episodes of loss of consciousness, vomiting, or ear, nose, or throat bleeding. The patient’s Glasgow coma scale (GCS) was E4V5M6 at the time of arrival at the emergency department. There were no blunt injuries to the chest or abdomen. An X-ray of the right shoulder was performed in posteroanterior view, which revealed a fracture of the mid-shaft of the right clavicle (Figure 1).

The patient was planned for ORIF with plating as an elective case. Baseline hematological and biochemical investigations were within the normal limits (Table 1). 

**Table 1:** Biochemical and hematological parameters, along with the institutional reference ranges

Investigation	Results	Normal range
Hemoglobin (Hb)	14.2 g/dL	12-16 g/dL
Total leucocyte count	6,800 cells per microliter	4000-11000 cells per microliter
Platelet count	2,54,000 per microliter	1,50,000-4,00,000 per microliter
Serum creatinine	1.1 mg/dL	0.7-1.3 mg/dL
Serum sodium	139 mmol/L	135 -145 mmol/L
Serum potassium	4.2 meq/L	3.5 -4.5 meq/L
Serum chloride	101 meq/L	96-106 meq/L
Prothrombin time	11.2 seconds	9-14 seconds
International normalized ratio (INR)	1.1	0.9-1.2
Activated partial prothrombin time (aPTT)	30.4 seconds	24-32 seconds

A pre-anesthetic checkup was done, and the patient was cleared for the proposed procedure under the American Society of Anaesthesiologists (ASA) physical status I. Routine premedication orders were advised as per the institutional protocol, which included tab. alprazolam 0.25 mg on the night before the surgery and tab. pantoprazole 40 mg on the night and the morning of the surgery. The procedure was planned under loco-regional plus sedation, and appropriate information and counseling were provided to the patient.

**Figure 2:**
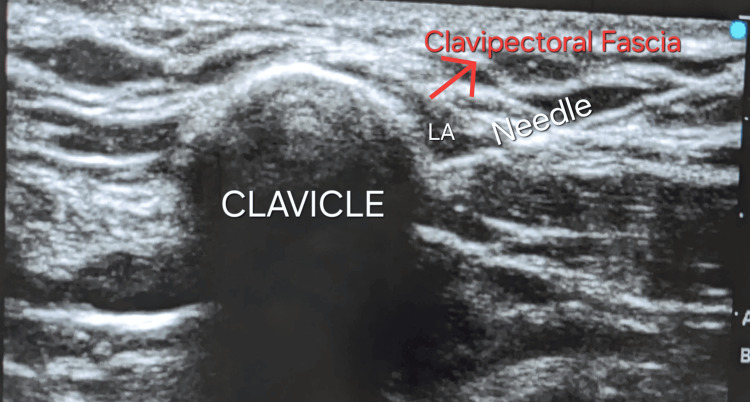
An ultrasound image shows the clavicle and the overlying clavipectoral fascia. A needle is seen piercing the fascia. LA: local anesthetic

On the day of the surgery, the patient was re-evaluated, and baseline vitals were recorded (heart rate: 84 beats per minute, blood pressure: 118/78 mmHg, and oxygen saturation: 99% on room air). The operation theater was prepared appropriately, and the patient was wheeled inside the theater. Monitors were attached as per the standard ASA guidelines, which included electrocardiogram (ECG), noninvasive blood pressure (NIBP), pulse oximetry, and end-tidal carbon dioxide monitoring. Midazolam injection (1 mg) was administered intravenously for anxiolysis. Under ultrasound guidance (SonoSite Edge II Fujifilm, Bothell, WA), a check scan of the desired area was performed. Under all aseptic precautions, the area of interest was painted and draped with sterile cloth. Lignocaine injection (2%, 4 mL) was infiltrated under the skin. Using a linear probe (6-13 MHz frequency), the clavipectoral fascial plane was identified, and needling was performed in the in-plane approach. Twenty mL of 0.5% bupivacaine was administered into the clavipectoral fascia, both proximal and distal to the fracture (Figure 2).

The onset of sensory block was assessed subjectively using a pin-prick test around the area of the fracture, the neck, and the upper arm. The onset of motor block was assessed by the ability of the patient to shrug the shoulders. A complete sensory and motor block was achieved within 20 minutes of the block. Dexmedetomidine injection was administered at a maintenance dose of 0.5 mcg/kg/hr. Oxygen supplementation was provided, and end-tidal CO_2_ was monitored throughout the case as the patient’s head and neck were under the drapes. 

The case lasted for 1.5 hours, during which the patient’s visual analog score (VAS) score and sedation score (using the Richmond Agitation Sedation Scale (RASS)) were monitored by lifting the drapes every 10 to 15 minutes and observing the patient. The sedation score was maintained between 0 and -1 during the surgery. In the first 24 hours postoperatively, the patient’s VAS score was below three, and he did not require any supplemental analgesics other than a paracetamol injection, which was scheduled at a dose of 1 gm every eight hours.

## Discussion

The clavicle (collarbone) is sigmoid-shaped and the only long bone that lies horizontally. It lies in the front part of the shoulder. It is articulated medially with the manubrium and laterally with the acromion process of the scapula. It plays an essential role in functional movement and acts as a brace for the shoulder, allowing weight to be transferred from the upper limb to the axial skeleton [5].

The innervation of the clavicle is complex, supplied by both the cervical and brachial plexuses. The clavicle is innervated by the spinal accessory nerve (cranial nerve XI), axillary nerve (C5-C6), nerve to subclavius (C5-C6), supraclavicular nerve (C3-C4), and lateral pectoral nerve (C5-C7) [6]. Multiple innervations of the clavicle make it difficult to choose one single anesthetic block technique for clavicle surgeries. 

Various studies have shown the use of different regional anesthesia techniques for clavicle surgery, among which interscalene cervical plexus block and superficial cervical plexus block, or their combination, have been used. Due to the increased time consumption required to administer two separate blocks and the adverse effects associated with the brachial plexus blocks, usually general anesthesia becomes the preferred choice for clavicle fixation surgeries [7].

Perioperative or surgical analgesia using a CFPB is effective. The CFPB, an alternative to the above regional techniques, is easier to perform, has the advantage of a single injection, is safer with the clavicle as the backstop, and is more lateral and superficial with no reported adverse events [8]. This block has similar analgesic efficacy to the brachial plexus block and avoids phrenic nerve palsy [9]. The block may also be beneficial to trauma patients with rib fractures or pneumothorax, in whom general anesthesia with positive pressure ventilation may be contraindicated. 

The integrity of the fascia plays an essential role in this block since it is a fascial plane block. The presence of a displaced or communited fracture can disrupt the fascial layer, leading to the improper spread of the drug. This may lead to an inadequate block [10]. In our case, though, the trauma might not have disrupted the fascial plane architecture, thereby not compromising the effect of the block. 

A combination of CFPB and light sedation (RASS 0 to -1) reduces the cost of administering general anesthesia and airway manipulation during laryngoscopy and intubation. Further, monitoring of the sensory and motor coverage could be better recognized before and after the procedure, which would be difficult in cases of general anesthesia. 

## Conclusions

The CFPB emerges as a promising and accessible regional anesthesia technique for effectively managing fractures of the clavicle. The use of CPFB along with supplemental analgesics like dexmedetomidine would suffice in managing the clavicular fractures. With its anatomically targeted approach and easier surgical steps, this block stands as a valuable addition to the armamentarium of pain management strategies. 
